# Role of Social Commerce Constructs and Social Presence as Moderator on Consumers' Buying Intentions During COVID-19

**DOI:** 10.3389/fpsyg.2022.772028

**Published:** 2022-02-09

**Authors:** Rao Muhammad Rashid, Abdul Hameed Pitafi, Muhammad Asif Qureshi, Anshuman Sharma

**Affiliations:** ^1^Department of Management Studies, Bahria University, Karachi, Pakistan; ^2^Department of Management Studies, Muhammad Ali Jinnah University, Karachi, Pakistan; ^3^Department of Computer Science, Sir Syed University of Engineering and Technology, Karachi, Pakistan; ^4^Department of Marketing, College of Business Administration, Ajman University, Ajman, United Arab Emirates

**Keywords:** social commerce constructs, social presence, social support, trust, COVID-19, buying intention

## Abstract

Social interactions through social commerce platforms empower consumers to share their personal experiences with others, but its role becomes more significant for societal protection during COVID-19. Numerous scholars have studied e-commerce extensively, but there is a lack of studies to identify social commerce characteristics to attract potential consumers during COVID-19. This study aims to examine the role of social commerce constructs by integrating social presence as a moderator in the model to explain consumers online shopping intentions in a Pakistani context, where lack of trust on the reliability and validity of comments from other consumers being considered the growing concern toward the success of social commerce. The quantitative data were collected from the respondents living in different cities of Pakistan. Most of the hypothesis supported and demonstrate the positive response from the Pakistani consumers having experience in shopping through social commerce platforms. The findings of this study will help scholars and managers to understand the attitude of Pakistani consumers.

## Introduction

Web 2.0 technologies enable social commerce to expand rapidly (Hajli et al., 2017; Mikalef et al., 2017). Social commerce can bring sociability to consumers through social environments, especially during pandemic (COVID-19) outbreaks, for example, websites for social interaction. Social commerce platforms provide different features such as ratings, reviews, tags, recommendations, referrals to buy online, and user profiles to share their experiences (Kim and Park, 2013), which provides more social support and develops a trustworthy environment for business through social commerce platforms (Shadkam and O'Hara, 2013), which can considerably impact the situation during pandemic (COVID-19) outbreak. Several consumers prefer to share their own experiences through social commerce platforms because users are indirect acquaintances or friends. This information sharing looks more accurate and real than sellers (Bai et al., 2015). Trustworthy reviews and ratings play a significant role in developing consumers' buying intentions and thus stimulate product sales, whereas social commerce constructs will enhance consumers' level of social support and trust in sellers. With the help of this knowledge, managers can provide services or marketing activities to improve the level of social support and trust, especially during COVID-19.

Earlier studies have documented that social commerce constructs have a considerable significant influence on social support and trust which sequentially affects their buying intentions (Hajli and Sims, 2015; Li, 2017). For example, Hajli (2015) found that social commerce constructs influence social support, the increasing prevalence of positive customer purchase intentions. In contrast, Li (2017) found that social commerce constructs significantly impacted consumers' trust in sellers, which affected their product recommendations. Consumers' trust in product recommendations has a significant effect on their buying intentions. On the other hand, social support and consumers' trust in sellers is their subjective experience, which can change over time or with perceived surroundings. Social presence is an experience of seeing others' existence in the medium that consumers observe from online podiums in the non-existence of face-to-face interaction between consumers and sellers, which has a significant influence on social support and trust (Jiang et al., 2019; Rashid et al., 2019, 2020). In the COVID-19 quarantine period, people have more time to spend on social commerce platforms, which could be a key source of anxiety and rumors; companies' social presence can reduce the effect of fake news and increase consumers' trust in sellers. Hence, social presence may moderate the link between social commerce constructs and social support, and consumers trust sellers. We can better understand social commerce constructs and their impact on social support and customer trust in sellers using social presence.

Considering the points mentioned above, this article investigates how social commerce constructs will enhance social support, consumers' trust in sellers, and how social presence moderates the association between social support and social commerce constructs, consumers' trust in sellers during pandemic COVID-19. By integrating social commerce constructs, social support theory, and social presence theory, we develop a conceptual model to explain how social commerce has a strong positive impact on social support and consumer's trust in sellers and how both of these factors influence customers' purchasing intentions in social commerce contexts. Social presence is a moderating construct among social commerce constructs and social support, consumers' trust in sellers.

This article makes some significant additions to the boundaries of the existing literature. First, evaluated with the quantitative valuations of social commerce constructs and present studies on the current aspects of social interactions, this article redefines the role of social commerce constructs in a Pakistani context, where deficit of trust on the authenticity and dependability of other customers' remarks being considered the emergent concern toward the accomplishment of social commerce. Second, it explores the moderating role of social presence between social commerce constructs and social support, consumers' trust in sellers, which describes how social commerce constructs and social presence rally on social support and consumers' trust in sellers during pandemic COVID-19. Third, this article studies the joint influence of social commerce constructs, social support, consumers' trust in sellers, and social presence on consumers' buying intentions in the social commerce environment during pandemic COVID-19.

The remainder manuscript is schematized in this way: the literature review throws light on the existing body of the literature, whereas section development of hypotheses discusses hypothesis development from the proposed model in detail. Section design or methodology lightens the process of questionnaire development, pilot study, and data collection, while sections data analysis, discussion, implications, and limitations and future research directions.

## Literature Review

### Social Commerce

Social commerce integrates social media technologies in e-commerce platforms, allows customers to communicate with one another and businesses through online groups, societies, and generates reviews, ratings, and recommendations. Previously, the management and initiation of interactions between consumers and businesses were expensive and impractical (Al-Adwan and Kokash, 2019). However, the advancement of social technology has added a social component to online buying, which results in a more sociable experience during the pandemic. Furthermore, these technologies enable organizations to initiate and manage social connections with customers in a cost-effective and manageable manner.

Social commerce is categorized into two classes; first class is based on traditional e-commerce sites which are product-oriented and offer one-way interaction from business to the customer, for example, Amazon, whereas the second category is based on the integration of the social aspects in e-commerce platforms which are customer-oriented and support two-way interactions (Huang and Benyoucef, 2013). Consumers can react to services or products on various podiums and speak with each other. Consumer participation is a significant feature of social commerce. Numerous businesses contend with others for better sales and reputation in social commerce. For instance, these social commerce platforms, Facebook, Instagram, and Twitter, provide several paths of C2C and B2C; both have connections with buyers and sellers. The study aims to discover whether or not social commerce constructs and social presence effectively moderate consumer's buying intentions in the social commerce environment; this study considers that the social interaction between consumers on social platforms may influence their buying decisions during pandemic COVID-19. By following the aim of this study, we have narrowed the scope of this study to the second category because the first category provides limited options for social interactions. Thus, a detailed study of social commerce can help us better understand this new way of doing business and guide practical and academic research during COVID-19.

### Buying Intentions

The progression of online buying improved rivalry in the online marketplace (Akroush and Al-Debei, 2015) and transformed the retail setting for consumers and retailers (Kühn and Petzer, 2018). Buyers had a better buying experience, and retailers have more customer support and engagement opportunities. Few studies focused on the factors influencing e-commerce websites and the characteristics of e-commerce sites. Factors studied include perceptions of web design, privacy based on adoption theory, customer service, and reliability (Kühn and Petzer, 2018); trust on the webpages and attitudes in the direction of the webpage based on the stimulus-organism-response framework and TPB (Wu et al., 2017). Added factors investigated were website trust and flow (Kühn and Petzer, 2018), store reputation, brand knowledge, size, risk, and personal, organizational belief (Dutta and Bhat, 2016). These current studies recommend stores and retailers to upsurge consumer acceptance of their stores and webpages to upsurge sales. Therefore, the earlier investigation has shown that effective online business involves a deep understanding of how these several factors affect customers' buying intentions.

Some studies have focused on online shopping behavior, especially in the context of the COVID-19 pandemic. For instance, Ozturk (2020) studied the effects of utilitarian and hedonic beliefs. Eti et al. (2021) examined the impact of social media. Jiaming et al. (2020) concentrated on social presence, fear appeal, and e-loyalty in buying in the pandemic. Along with empirical examination, Zwanka and Buff (2021) presented a context of the buyer behavior shifts, and Sheth (2020) suggested how COVID-19 inclined consumers' behaviors. Some research on consumers buying through social commerce available in COVID-19 has focused on a particular product type or industry. However, previous studies have not examined pandemic concerns as a precursor to social commerce constructs, social presence, social support, and consumers' trust.

### Social Commerce Constructs

Social commerce constructs are the built-in features of a website that enable consumers to interact with other consumers and refer, rate, comment, and shop products (Hajli, 2013). These features can enhance sociability among consumers (Hajli and Sims, 2015), which can significantly influence the situation during pandemic COVID-19. For example, characteristics that support social buying activities are forums, blogs, videos, reviews, recommendations, and social networks (Kim and Park, 2013; Ng, 2013). This study considers reviews, ratings, referrals, and recommendations as to the critical social commerce constructs. Reviews and ratings are visible to all and defined as the set of features provided by the social commerce websites that enable consumers to share feedback with other consumers (Shadkam and O'Hara, 2013). In contrast, referrals and recommendations are personalized online social activities that allow consumers to share information with peers in making buying decisions (Kim and Park, 2013; Rashid et al., 2019). Furthermore, social commerce constructs enable sellers to improve their social presence, social support with peers on social commerce podiums, and the development of trust, which enhances consumers' buying intentions, especially during a pandemic.

### Social Support

Social support is care or support offered by a group of people or a group of people and sellers. The social support theory is used frequently to illustrate how social interactions influence consumers' emotions and behaviors (Cohen and Wills, 1985; Cohen and McKay, 2020).

In the social commerce context, social support can be separated into two groups: informational support “offering guidance or advice for the benefit of solving problems, and emotional support primarily interested in communicating with customers about feelings such as empathy, compassion, and care”—online social interactions spawned through communication mediated by computer and dependent on online interactions by using social podiums (Caplan, 1974). Hence, practical social support is gauged as elusive, and it requires informational and emotional help (Madjar, 2008).

The consumer's sense of closeness is further enhanced by social interaction with online sellers, especially in a pandemic situation. In an online context, strong feelings strengthen social bonds between customers and motivate them to share product or service information or feedback. In this light, these social contacts may aid in developing a relationship between buyers and sellers. Consumers will have more positive opinions if a high level of social support is recognized (Hassanein and Head, 2007). As a result, in a social commerce environment, social support plays an integral role in developing consumers' buying intentions during the pandemic.

### Consumers' Trust in Sellers

Trust is when you have the confidence that a promise will be kept to the last agreement's specifications. Because virtual purchasing environments make up such a significant part of today's marketplace, it is vital in the online market. If you do not have confidence in your products, you will never build an online selling business. McKnight and Chervany (2001) segregated trust into different categories; trusting intentions (individual consumers' intentions toward the behavior of online sellers) and trusting beliefs (trust of consumers in online sellers or podiums).

Online sellers use different practices to improve consumers' sympathy and keep a reliable link with them, that is, virtual reality buying or through visuals. These skills shape and enhance social interactions between consumers and help consumers better understand online sellers. Contemporary literature argued the importance of trust in consumers' buying intentions in the social commerce environment (Rashid et al., 2020); however, during COVID-19, people stay at home most of the time; in this scenario, trust is important in social commerce interactions and purchases. A better understanding of consumer's trust in social aspects and buying intentions is noteworthy.

### Social Presence

Social presence is the feeling of being present in social media, whether online or in the real-world Short et al. (1976). Online buying, which lacks face-to-face interactions between sellers and customers, demands social presence (Cyr et al., 2007; Jiang et al., 2019). The study findings suggest that media richness is connected to perceived distance and intimacy levels, influencing how frequently participants see each other. Researchers previously relied on the social presence theory to study social commerce purchasing. Incorporating human-like behaviors and gestures in a product design demonstrates competence in the ability to transfer social signals, such as persuasion (Pavlou et al., 2007), virtual agents (Hess et al., 2009), and socially-rich messages (Gefen and Straub, 2004). Social presence is an integral part of the online buying experience in media entertainment (Tamborini et al., 2010; Shin et al., 2019). In addition, for businesses involved in social commerce, social presence enables customers to access more incredible information and interact with others, resulting in better relationships and buying decisions.

## Hypothesis Development

**Figure 1:**
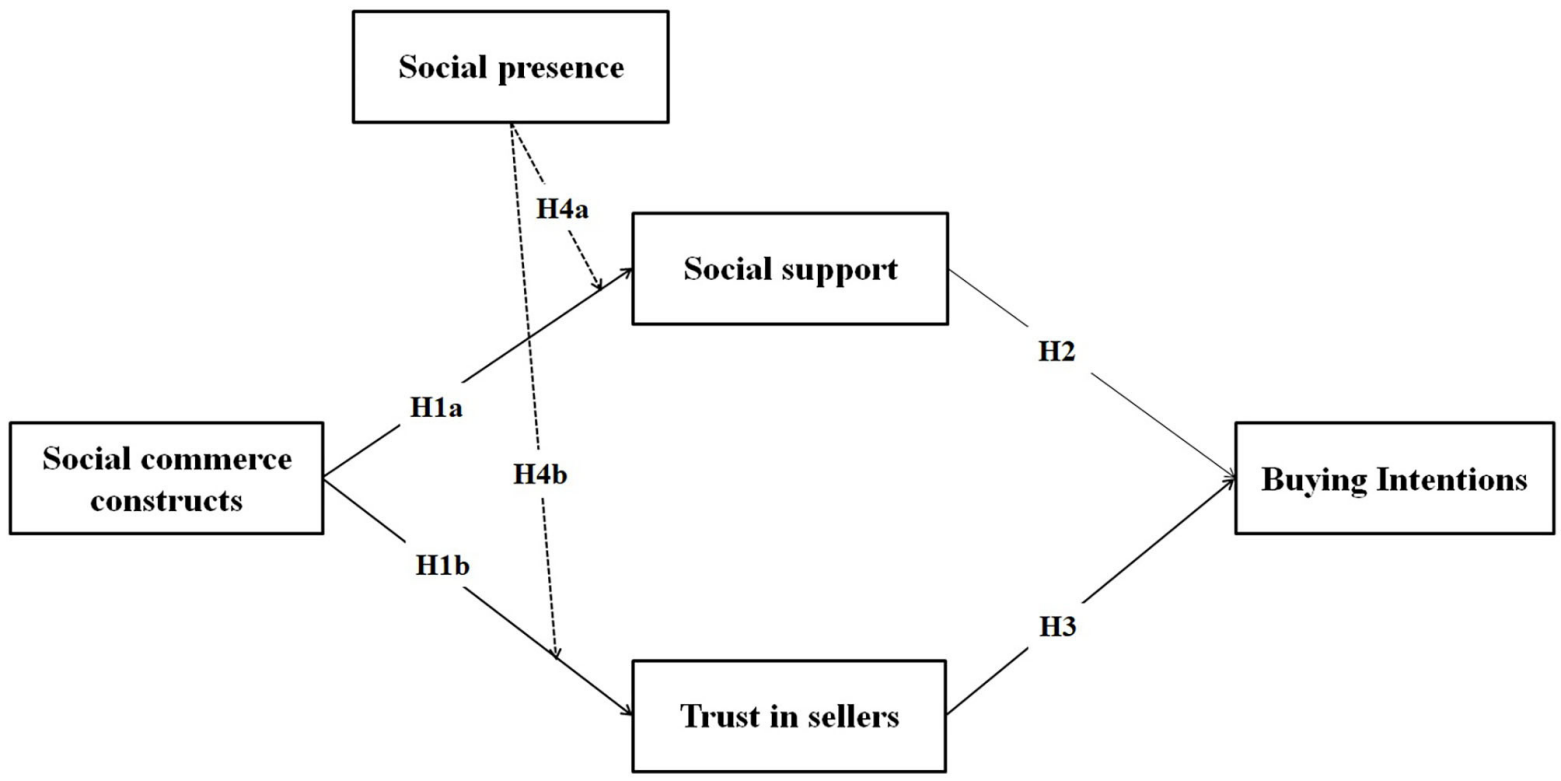
Proposed study model.

The social commerce construct, social support, trust, social presence, and purchasing intention are all included in the research model for this study. Two hypotheses (H2 and H3) seek to discover whether customers' buying intentions are related to their concepts. Social commerce will impact social support and trust through hypotheses H1a and H1b. Furthermore, H4a-b looks into the relationship between social commerce constructs, social support, and consumer's trust in sellers. The study presents a model based on these literature-supported assumptions, and Figure 1 depicts the study's model.

### Social Commerce Construct, Social Support

Social commerce constructs provide social support to consumers and share personal experiences, reviews, ratings, and referral services and products. Getting information from others might help consumers solve their buying problems and offer them social support. During pandemics, ratings and reviews from unknown consumers provide social support for other consumers because those reviews and ratings are considered more credible (Li et al., 2021b). Hence, during pandemics such as COVID-19, consumers can exchange information and interact with others through referrals, recommendations, reviews, and ratings on social commerce platforms, leading toward love, care, and support from peers. Based on these arguments, we hypothesize that:

**H1a**. Social commerce constructs will positively influence social support during COVID-19.

### Social Commerce Construct, Trust in Sellers

Businesses use different platforms to communicate with consumers and enable consumers to interact with each other in pandemics. They can thus utilize social commerce platforms to improve sales. These platforms provide a prospect for consumers to gain more knowledge through social interactions, exchange information, review, and recommend a product or service, which sequentially increases their level of trust in sellers (Li et al., 2021a). During COVID-19, social commerce constructs such as reviews, ratings, referrals, and recommendations can play a vital role in developing consumers' trust in sellers (Li et al., 2021b). Thus, we hypothesize the following:

**H1b**. Social commerce constructs will positively influence consumers' trust in sellers during COVID-19.

### Social Support, Buying Intention

Social support is vital for improving social interactions and for motivating people to reciprocate. Consumers are more likely to share products, services, and experience information with friends and family when they reciprocate motivation (Kurt et al., 2021). Consumers who have had a positive social support experience are more inclined to share purchasing information with others in pandemics. As a result, sharing purchasing information with other customers fosters a positive environment (Kurt et al., 2021); this leads to more stable consumer's purchasing intentions in pandemics such as the COVID-19. Thus, we suggest the following hypothesis:

**H2**. Social support will positively influence consumers' buying intentions in a social commerce environment during COVID-19.

### Trust in Seller, Buying Intention

From the customer's perspective, during pandemics, the concept of social commerce describes consumers' desire to engage in online shopping using social platforms as “on-the-ground buying *via* social podiums.” When people share information about products and services in online forums and communities, the resulting connections promote trust and improved online purchasing intentions. Kurt et al. (2021) explained that trust could reduce behavioral uncertainty when consumers buy on sellers' web pages, decreasing psychological blockades related to online buying. Hence, we believe that consumers' trust in sellers increases their buying intentions through social commerce in COVID-19. Therefore, we hypothesize the following:

**H3**. Consumers' trust in sellers will positively influence their buying intentions in the social commerce environment during COVID-19.

### Social Presence as a Moderator

Social commerce podiums permit consumers to each other. Social commerce constructs can amplify support among consumers and firms. Increased social presence helps to improve the level of social support between consumers during COVID-19. During COVID-19, businesses have a higher social presence, social commerce construct of social support is strengthened. For example, users in social commerce platforms provide a lot of information through reviews, ratings, referrals, recommendations, etc. Social commerce platforms' notes feature can simply this way of firms' social presence, which can more easily provide support by integrating artificial intelligence (Rashid et al., 2020). This will positively influence social support. Hence, we make the following hypothesis:

**H4a**. Social presence positively moderates the association between social commerce constructs and social support during COVID-19.

During pandemics such as COVID-19, businesses can encourage customers to provide feedback through reviews and ratings, whereas referrals and recommendations empower consumers to share information related to product quality standards with peers. This support from consumers brings a sense of imminence and trust in sellers (Zhong et al., 2021). Suppose businesses can offer more support in the route of the social commerce platforms for interaction between sellers and consumers during COVID-19. In that case, customers may receive more support and feel the actual presence of a big business. For instance, businesses' social presence may be essential for customers who have similar product concerns in social podiums and cannot identify the grounds of quality issues during COVID-19. Thus, we suggest the following hypothesis:

**H4b**. Social presence positively moderates the association between social commerce constructs and consumers' trust in sellers during COVID-19.

## Methods

### Measurements

All of the items were found within the existing body of research, except slight modifications, from “strongly disagree” (1) to “strongly agree” (7). The content and style of the items were tailored to fit in with the social commerce environment. We also asked questions regarding demographics such as gender, age, and level of education. Information regarding the questionnaire was revealed through self-administration on docs.google.com, and also through the Internet.

### Data

We conducted the pilot study with 54 responses, obtained detailed comments on the structure and content of questions, and made changes accordingly for the final study. Participants of the pilot study were asked to give suggestions and provide feedback on statements not clear to them. Participants attempted all questions by following the given instructions. The significant changes were made in the items of social presence, and buying intention, as they generated ambiguity, and participants were facing problems in understanding the questions. Therefore, by following the study context (i.e., social commerce), the items were tailored by several suggestions regarding the phrasing and structure of the questionnaire. The data collected in the pilot study stage were not included in the main study survey.

Due to COVID-19, online survey through scholar.google.com during April 2021 and May 2021 was conducted to test the hypothesis in this study. Participants of this study were those who possess the experience of buying through social commerce platforms. The data were collected from social commerce consumers who had experienced presence, support, reviews, ratings, referrals, and recommendations. We did the shortlisting of answers to each question. Wrong answers, for example, having similar answers for all questions, were removed from our sample. In total 334, people finished the questionnaire successfully, which can easily be considered as a sufficient sample for this study (Hsia et al., 2014). Table 1 shows demographic details. SPSS25 was used for descriptive statistics and correlation. Structural equation modeling (SEM) was performed using AMOS24, which is documented as a robust statistical tool and is widely used in CFA and SEM. The data screening phase included analysis of normality, multivariate outliers, missing values, multicollinearity, correlations, and descriptive statistics.

**Table 1:** Demographics.

	***N***	**Percentage**
**Gender**		
Male	226	67.7
Female	108	32.3
**Age**		
18–28 years old	166	49.7
29–38 years old	150	44.9
39–49 years old	18	5.4
**Education of respondents**		
Under graduate	48	14.4
Bachelor	230	68.9
Master	56	16.8
**Online buying frequency**		
Numerous times in a week	70	21.0
Numerous times in a month	50	15.0
Once or twice a week	76	22.8
Once or twice a month	138	41.3

## Analysis Results

### Measurement Model

To examine the measurement model in this study, we used confirmatory factor analysis. Specifically, we looked at the convergence, discriminant, and content validities. Convergent validity was measured by examining the values of composite reliability (CR), average variance extracted (AVE), and factor loadings, where convergent validity measures the extent to which a construct is linked with other constructs in the suggested study model. The minimum threshold levels for Cronbach's alpha, CR, and AVE are 0.70, 0.70, and 0.50, respectively (Fornell and Larcker, 1981). Fornell and Larcker (1981) suggested that composite reliability and Cronbach's alpha are the two trustworthy statistical techniques for determining construct reliability, which is considered suitable if CR and α-values are above 0.70. The content validity was also measured through a pilot study. Table 2 shows that the factor loadings of all proposed items are higher than the threshold value of 0.70. Additionally, discriminant validity, the value of innercorrelation between components is shown in Table 3, was below the square root of AVE each factor (Fornell and Larcker, 1981).

**Table 2:** CFA results.

**Construct**	**Loading**	**CA**	**CR**	**AVE**
**Social commerce constructs (Pagani and Mirabello**, **2011**, **2012****)**				
I am interested in reviews and ratings from other users.	0.764	0.92	0.93	**0.66**
The members who review and rate products/services on this platform are fairly knowledgeable about the platforms topics.	0.848			
A key motive I like this site is the reviews and ratings from other users.	0.899			
I am interested in reading referrals from other users.	0.836			
The members who refer products on this page have fair knowledge about the products available on this page.	0.769			
A key reason I like this site is the recommendation from other users.	0.825			
This site does a good job of getting its visitors to make referrals.	0.716			
**Social presence (Rashid et al.**, **2020****)**				
There is a sense of sociability in social commerce platforms.	0.803	0.88	0.87	0.58
There is a sense of human warmth in social commerce platforms.	0.677			
There is a sense of personalness in social commerce platforms.	0.933			
This website has eye-catching commodity images on the home page.	0.726			
I can sense others who provide information about the seller.	0.646			
**Social support (Jiang et al.**, **2019****)**				
When encountered with complexities, my friends on the social platforms comforted and encouraged me.	0.616	0.82	0.72	0.52
My friends on social platforms would offer suggestions when I needed support.	0.609			
When I faced a difficulty, my friends on social platforms would assist me conquer the difficulty.	0.658			
**Consumers' trust in sellers (Kim and Park**, **2013****)**				
This seller would be trustworthy.	0.825	0.88	0.89	0.66
This seller site could be trusted.	0.832			
This seller would be honest and truthful to me.	0.815			
Trust in seller positively influences my intention to use social commerce platforms for buying.	0.778			
**Buying intention (Li**, **2017****)**				
I am very likely to buy/hire the product/service from same seller.	0.621	0.83	0.79	0.54
I would consider buying the product/services from the same seller and platform in the future.	0.699			
I intend to buy the product/service from the seller.	0.717			

*CA, Cronbach's alpha; CR, composite reliability; AVE, average variance extracted; MSV, maximum shared variance; AVE, average variance extracted; ASV, average shared variance; discriminant validity = AVE > MSV*.

**Table 3:** Means, standard deviation, and correlations.

**Variable**	***M***	**SD**	**1**	**2**	**3**	**4**	**5**	**6**	**7**	**8**	**9**
1. Social commerce constructs	3.69	0.80	**0.81**								
2. Social presence	3.69	0.95	0.57^**^	**0.76**							
3. Social support	4.08	0.58	0.20^**^	0.16^**^	**0.72**						
4. Consumers' trust in sellers	3.65	0.75	0.35^**^	0.37^**^	0.37^**^	**0.81**					
5. Buying intention	4.00	0.64	0.25^**^	0.31^**^	0.50^**^	0.24^**^	**0.73**				
6. Online buying frequency	**NA**	**NA**	−0.12^*^	−0.24^**^	−0.01	−0.25^**^	−0.03	**NA**			
7. Education	**NA**	**NA**	0.11^*^	0.13^*^	0.08	0.15^**^	−0.06	−0.49^**^	**NA**		
8. Age	**NA**	**NA**	−0.08	−0.18^**^	−0.06	−0.18	−0.02	0.67^**^	−0.35^**^	**NA**	
9. Gender	**NA**	**NA**	0.08	0.08	0.00	0.13^*^	−0.10^*^	−0.08	−0.06	−0.10^*^	**NA**

* *p < 0.05*.

** *p < 0.001. Boldface numbers are the square root of the AVE*.

### Common Method Bias

Researchers suggested a likelihood of common method bias in the data set when data are collected through a single source (Podsakoff et al., 2012). By ensuring the validations of Podsakoff et al. (2003), numerous remedies were considered before and the data collection. First, questionnaire translation was pretested and basic corrections were made before starting data collection; second, we guaranteed respondents their privacy; third, the well-established sale was used to shun any ambiguity. In addition, as Podsakoff et al. (2003) proposed, to check common method bias was an issue or not, we have selected Harman's single-factor test. The findings show a variance value less than the cutoff value of 25.08% (i.e., 50%). Moreover, Table 3 shows that the innercorrelation between all constructs is below 0.90 (Pavlou and El Sawy, 2006). These results indicate that there is no significant concern of common method biasness in this study. Table 4 shows Comparison measure model and structural model.

**Table 4:** Comparison measure model and structural model.

**Absolute fit measures**				**Incremental fit measures**		**Parsimonious fit measures**		
**Model**	***X*^2^/DF**	**SRMR**	**RMSEA**	**NFI**	**PNFI**	**CFI**	**IFI**	**TLI**
MM	3.54	0.08	0.08	0.85	0.81	0.90	0.89	0.87
SEM	4.91	0.08	0.08	0.84	0.80	0.89	0.89	0.86

### Hypothesis Testing

This research validates that social commerce constructs significantly and positively predict social support (β = 0.18, *t* = 3.98, *p* < 0.01). Therefore, H1a is validated. Further, social commerce constructs significantly and positively related to trust in sellers (β = 0.38, *t* = 6.71, *p* < 0.01). Hence, H1b is supported. In addition, social support is positively related to consumers' buying intentions in social commerce environment with (β = 0.39, *t* = 6.89, *p* < 0.001), supporting H2. Moreover, results from this study reveal that trust in sellers is positively related to consumers' buying intentions in the social commerce environment (β = 0.15, *t* = 3.28, *p* < 0.01). Thus, H3 is confirmed. The results of all hypotheses testing are shown in Table 5.

**Table 5:** Hypothesis testing.

	**Path**	**Standard coefficient**	***t*-value**	**Result**
H1a	Social commerce construct to Social Support	0.18	3.98^**^	Supported
H1b	Social commerce construct to trust in sellers	0.38	6.71^**^	Supported
H2	Social support to buying intention	0.39	6.89^***^	Supported
H3	Trust in sellers to buying intention	0.15	3.28^**^	Supported
	Online shopping frequency to shopping intention	−0.08	−2.89	Insignificant
	Education to shopping intention	−0.11	−3.91	Insignificant
	Age to shopping intention	0.03	1.36	Insignificant
	Gender to shopping intention	−0.07	−2.66	Insignificant

* p < 0.05.

** *p < 0.001*.

*** *P < 0.0001*.

**Figure 2:**
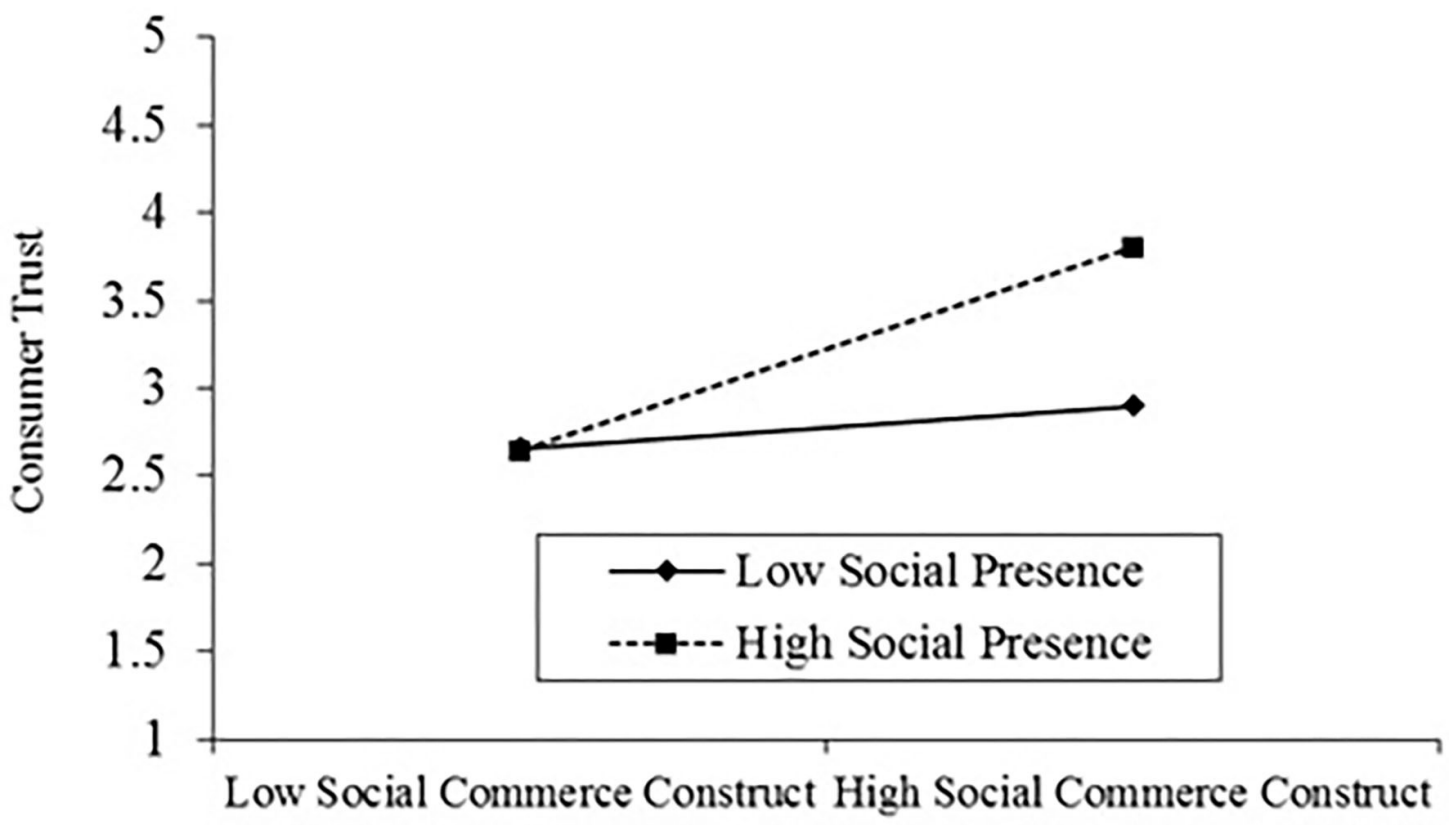
Showing moderation.

Finally, the outcomes show that social presence moderates the association among social commerce constructs and trust in sellers (β = 0.23, *t* = 3.44, *p* < 0.01), supports H4a. Similarly, our results reveal that social presence is not moderating the association between social commerce constructs and social support (β = 0.10, *t* = 1.48^ns^). Thus, H4b is not supported. To provide further evidence validating the moderating effects proposed in H4a and H4b, these results' slopes were plotted. The outcomes of moderation are shown in Table 6. Figure 2 shows the results of moderation.

**Table 6:** Moderation analysis.

	***B***	**SE**	***t***	***R* ^2^**
Outcome: consumer's trust				0.25
Constant:	−0.08	0.04	−1.93	
Social commerce construct	0.35	0.07	4.56^**^	
Social presence	0.23	0.06	3.44^**^	
Social science construct * social presence	0.22	0.06	3.66^**^	
Online shopping frequency	−0.05	0.04	0.31	
Education	−0.08	0.07	−1.11	
Age	−0.04	0.07	0.57	
Gender	0.09	0.03	2.42	
Outcome: social support				0.14
Constant:	−0.11	0.05	−2.23	
Social commerce construct	0.35	0.07	4.95^**^	
Social presence	0.10	0.06	1.48	
Social science construct * social presence	0.28	0.05	5.40^**^	
Online buying frequency	0.17	0.07	1.94	
Education	0.05	0.06	0.85	
Age	−0.14	0.07	0.85	
Gender	0.01	0.04	0.27	

*p < 0.05.

** *p < 0.01*.

## Discussion and Implications

### Discussion

Social commerce cannot thrive if you do not understand consumers' process when making purchasing decisions (Tajvidi et al., 2017), as it becomes more important, especially during COVID-19. Thus, researchers and practitioners need to investigate the primary causes of buying intention in social commerce environments. Results from prior investigations on buying intention have largely failed to integrate social commerce constructs, social support, consumers' trust in sellers, and social presence; thus, no holistic picture of the formation mechanism of buying intentions in social commerce environment is drawn. Based on the literature backed model, this study examined the role of social commerce constructs, social support, and consumers' trust in sellers by integrating social presence as a moderator in the study model to describe consumers' buying intention in the social commerce environment. Our findings extend the existing literature on social commerce constructs, social support, and trust (Lee and Kwon, 2011; Hajli et al., 2017). Additionally, we extend existing works (Tajvidi et al., 2017) on social presence, and investigative research using moderation strategies uncovered how social presence influences social commerce, social support, and trust constructs.

First, the statistical results of this study validate that social commerce constructs enhance consumers' perception of social support in a pandemic situation. This result is consistent with earlier studies (Hajli and Sims, 2015). Consumers can improve their relationships with other consumers through reviews, ratings, referrals, and recommendations and increase familiarity with social commerce platforms. Moreover, when consumers are more responsive to social commerce platforms, they trust other referrals and recommendations, reducing rumors and anxiety among consumers during pandemic COVID-19. This finding is aligned with earlier studies (Zhang et al., 2014), that enhanced social interactions improve consumers' trust in sellers.

Second, the findings of this study confirm the relationship between social support and consumers' buying intentions in a social commerce environment. This result is in line with earlier findings of Tajvidi et al. (2017). Consumers seek prepurchase product information from various sources (e.g., blogs and peers), and they trust peer recommendations more than business information. Consumers frequently share their knowledge experiences with other consumers over social contact, thereby assisting them in making purchasing decisions, which has an ample impact on their buying intentions.

Further, the relevance of the data is determined by the statistical results of trust in sellers on consumers' buying intentions during pandemic COVID-19. This finding supports the results of Hajli et al. (2017), which shows that consumers' buying intentions in social commerce environments are considerably inclined to trust sellers. The value of the coefficient of determination (*R*^2^) for social support is 0.626, for trust in sellers is 0.626, and for buying intentions is 0.464. Approximately 62.6%, 0.626 of the change in social support and trust in sellers is explained by social commerce constructs, respectively. The sellers' social support and trust variation describe 46.4% of the shift in buying intentions. This demonstrates that the recommended model is well fitting.

In conclusion, the outcomes indicate that social presence significantly moderates the association between social support and social commerce constructs. In the COVID-19 quarantine period, people have more time to spend on social commerce platforms, so a higher level of social presence can substantially develop the level of information available to consumers through peers, family, friends, etc. This promotion of presence can increase the level of support available from others and also review ratings, referrals, and recommendations (Rashid et al., 2020). So, the outcomes recommend that a higher level of social presence is vital between social commerce constructs and social support. However, contrary to our hypothesis, social presence does not moderate the association between social commerce components and customer trust in sellers. One probable description is that social presence has a mild emphasis on the association between social commerce structures and consumer's trust in sellers. Consumer reviews and suggestions might be misleading and prejudiced at times. The enhancement of social presence may not prop up the amateur reviews and recommendations and decrease the degree of reviews and recommendations asymmetry.

### Theoretical Implications

The following theoretical illumination comes from the study findings. First, this study significantly contributes to the existing body of work by theorizing the role of social commerce constructs (i.e., reviews, ratings, referrals, and recommendations), social presence and support theory, and other ramifications of consumers' purchasing intentions, which offers a new dimension in research on social commerce. The study model (Figure 1) and statistical results shed light on the application of social commerce constructs, social support, social presence, consumers' trust in sellers in developing the broader knowledge of target customers' buying intentions in social commerce environments and its consequences, especially in COVID-19 outbreak. Several earlier pieces of research on social commerce have emphasized other facets, for instance, as an antecedent to commercial or social need (Hew et al., 2018), antecedent to design social podiums (Mikalef et al., 2017), antecedent to virtual client encounters and technological environs (Zhang et al., 2014), and antecedent to information dimensions people, technology, and management. This study differs from previous research studies by establishing its significant influence on social commerce constructs, social support, social presence, consumers' trust in sellers being used as a motivator in this way. Scholars will be able to investigate consumers' behavioral intentions in a new way in the context of social commerce.

Second, this study significantly contributes to the existing literature by theorizing the moderating role of social presence on the relationship between social support, consumers' trust in sellers, and consumers' buying intentions. The study findings confirm our argument that a higher level of social presence will strengthen the influence of social support and consumers' trust in sellers on consumers' buying intentions (Jiang et al., 2019).

Finally, this research emphasizes the significance of social presence through social interaction in social commerce platforms Web 2.0 to help individuals in buying decisions. These interactions offer different opportunities to businesses, for example, to modify their business strategies to meet the current social environment.

### Practical Implications

This study has a significant practical basis on the preceding conclusions and illumination during pandemic outbreaks. First, global and national companies will build strategies to meet consumer wants and improve their brand reputation if they understand the elements that influence consumers' purchasing intentions. Firms can further increase the level of social presence, reviews, ratings, and product-related information to develop consumers' trust. For example, using social settings such as Chatbots, sellers can provide sociability to customers.

Second, social commerce constructs such as review, ratings, referrals, and recommendations play a significant role in strengthening relationships and customer–business interactions during pandemic outbreaks. Social commerce platforms not only provide online communication to consumers but also support them in social interaction. Hence, sellers must understand and design appropriate strategies to develop particular features of social commerce platforms to target a specific consumer segment. Managers can attract consumers to share information or generate content to improve profits generated by enticing new customers.

Finally, this study proposes that firms design social commerce platforms to increase consumers' trust in sellers, social interaction between consumers through reviews, personal messages, recommendations, social support, and social presence during pandemic outbreaks. These social commerce platforms can offer prospects to businesses and people to interact with one another, increasing consumers' level of trust in sellers and influencing their buying intentions. Fourth, the findings of this investigation suggest that social presence strengthens consumers' intentions to buy on social media networks. Businesses, for example, can create social media sites to show more their presence through social interaction opportunities because coordination and communication improve the level of social presence.

## Limitations and Future Scope

The findings of this research were found significant and enlarged the scope of the existing knowledge about consumers' buying intentions in the social commerce environment, but it is not without limitations. First, the location of participants in this study limits the generalization of this study. Researchers could enlarge the scope of the target audience as it is no longer just zoomers who are active users of social commerce. Additionally, analysis of other demographic constructs and behaviors should be confirmed in future studies. Second, this research focuses on the moderating effect of social presence to suggest managers and scholars. This study opted for a few demographics as control variables, but other prospective constructs can influence consumers' buying intentions. For instance, content, design, message, media experience, etc., are prospective constructs influencing consumers' buying intentions. Future research should include more constructs to define better the role personal factors play in purchases. Additionally, future studies can also check other relationships as well. Finally, the variables were measured at a particular point in time. The longitudinal approach may be used in future investigations to corroborate the findings.

## Data Availability Statement

The original contributions presented in the study are included in the article/supplementary material, further inquiries can be directed to the corresponding author.

## Author Contributions

All authors listed have made a substantial, direct, and intellectual contribution to the work and approved it for publication.

## Conflict of Interest

The authors declare that the research was conducted in the absence of any commercial or financial relationships that could be construed as a potential conflict of interest.

## Publisher's Note

All claims expressed in this article are solely those of the authors and do not necessarily represent those of their affiliated organizations, or those of the publisher, the editors and the reviewers. Any product that may be evaluated in this article, or claim that may be made by its manufacturer, is not guaranteed or endorsed by the publisher.
